# Mindfulness-Based Cognitive Behavioral Therapy as an Adjunct Treatment of Attention Deficit Hyperactivity Disorder in Young Adults: A Literature Review

**DOI:** 10.7759/cureus.1269

**Published:** 2017-05-23

**Authors:** Muhammad Aadil, Rosario M Cosme, Jonathan Chernaik

**Affiliations:** 1 Department of Psychiatry and Mental Health, Rush University Medical Center; 2 Child Psychiatry, Rush University Medical Center

**Keywords:** attention deficit hyperactivity disorder, mindfulness based cbt, adults, psychiatry

## Abstract

Attention deficit hyperactivity disorder (ADHD) is a childhood-onset neurological disorder that often continues into adult age. Stimulants medication are the mainstay of treatment, however, in the recent years, there has been a lot of studies conducted to understand the effectiveness and feasibility of mindfulness-based cognitive behavioral therapy for treatment of attention deficit hyperactivity disorder in children and adults. In this article, we have reviewed 17 articles to look for the beneficial effects of such therapy in adults. Overall, we found that there is a clear beneficial effect of such therapies, especially when used in adjunct with stimulant medication and may increase overall compliance. For better understanding, we suggest that large, well-designed studies should be conducted with robust strategies, allowing more comparison studies with the better analytical outcome.

## Introduction and background

Attention deficit hyperactivity disorder (ADHD) is a disorder of neurodevelopment in children with high persistence into adulthood. ADHD manifests with high levels of inattention, impulsiveness, and hyperactivity. This condition affects social, academic and occupational functioning. Sign and symptoms of adult ADHD include difficulty prioritizing, organizing, and completing tasks, variable attention to detail, hyperactivity, and reduced impulse control. These deficits often lead to adverse psychosocial outcomes including academic failure, dropping out of school, unintended pregnancy, sexually transmitted infections (STI) exposure, and criminal behavior (e.g.: increased risk of traffic violations). Furthermore, such individuals have lower levels of educational and occupational achievement [1]. Hyperactivity tends to be a less salient feature in adults, but impulsivity and low frustration tolerance are often present to varying degrees [2]. In addition to this, ADHD patients often have one or more comorbid conditions including neurodevelopmental disorders (like dyslexia or autism) and psychiatric disorders (like anxiety or oppositional defiant disorder) [1]. This article will review treatment options for ADHD in adults and about practical and feasible mindfulness-based approaches in treating these symptoms. Current estimated prevalence of adult ADHD is 4.4% [3]. There have been many limitations in the past for the diagnosis of ADHD. The diagnostic and statistical manual (DSM-4) criteria were constructed with children in mind and offered only limited guidance regarding diagnosis in adults. As a result of relaxing strict age restrictions in DSM-5, estimates of prevalence based on the earlier criteria such as the one cited above should be considered conservative at best. Sixty-five percent of individuals diagnosed with ADHD in childhood continue to experience impairing symptoms at the age of 25 years [1, 4]. A study conducted in Australia comprising of 2179 individuals diagnosed with ADHD and aged 47–54 years (mean age 50.7±1.5 years) showed that inattentive symptoms were more significant compared to hyperactive symptoms (when depressive and anxiety symptoms were controlled) [5]. The inattentive symptoms are most common cause of functional impairment in adult ADHD [6]. The DSM-5 also notes that hyperactive symptoms become comparatively less evident compared to inattentive symptoms and difficulties persist with restlessness, abstraction, poor planning, and impulsivity [7]. A cross-sectional evaluation using the DSM-5 ADHD criteria was carried out in 3574 individuals from the study of 1982 Pelotas Birth Cohort to check the prevalence of ADHD beyond young adulthood [8]. ADHD prevalence rates in this study were found to be 2.1% for DSM-5 ADHD criteria and 5.8% for ADHD, disregarding age-of-onset criterion [8].

Given these, the fifth edition of the Diagnostic and Statistical Manual was released in May 2013 and replaced the previous version, the text revision of the fourth edition (DSM-4). DSM-5 included significant changes in the diagnostic criteria for ADHD and increased the guidance for diagnosing ADHD in adults: (1) Symptoms can now occur at age of 12 years rather than by age six years; (2) Several symptoms now need to be present in more than one setting, than just causing some impairment in more than one setting; (3) New descriptions were added to show the symptoms that might look at older ages; and (4) for adults and adolescents age of 17 years or older, only five symptoms are needed instead of six for younger children [7].

The standard of care for adults has evolved primarily from studies in children and the medications used in adults are the same as those employed in children and adolescents with ADHD. For adolescents of 12–18 years of age, the primary care clinician should prescribe Food and Drug Administration (FDA) approved medications for ADHD, with the assent of the adolescent and may prescribe behavior therapy as a treatment for ADHD, although preferably both medication and behavior therapy should be used together [9]. FDA-approved medications for ADHD in adults include amphetamine mixed salts, atomoxetine, dexmethylphenidate, lisdexamfetamine and methylphenidate [10]. Methylphenidate and amphetamine salts are the stimulant drugs of choice for ADHD treatment. The pharmacological effects of amphetamines occur because of increased presynaptic release of dopamine and other biogenic amines in the brain. Methylphenidate inhibits the reuptake of dopamine and norepinephrine. Lisdexamfetamine (prodrug of dextroamphetamine) has decreased abuse potential. Atomoxetine, a selective norepinephrine reuptake inhibitor is an alternative non-stimulant drug for ADHD but it is less efficacious than stimulants. Stimulants are safe, but weight loss, insomnia, headache, and anorexia are the most common adverse effects. They also carry a significant risk of abuse and dependence [11]. Although, pharmacological treatment has thus shown to be effective in reducing ADHD symptoms, about 10-30 % of adults with ADHD do not adequately respond to stimulants and others choose to discontinue stimulant treatment due to adverse effects like decreased appetite, dry mouth, tension, or jitteriness [12].

As such, there are a number of reasons for pursuing alternative or adjunctive treatments for ADHD in adults: some patients experience side effects of the stimulant medications use; others experience only a 30% reduction in symptoms; such stimulants are less effective in adults than in children and some patients are not willing to undergo pharmacological treatment. Because many patients undergoing pharmacotherapy still experience functional deficits related to decreased self-monitoring, inattention and mood disturbance. Interventions such as mindful awareness practices that directly address these problems could be used as adjuvant treatments [13].

## Review

Mindfulness-based interventions are a type of cognitive training involving various strategies to improve attention, affective self-regulation, tranquility and better quality of life in a healthy population [14]. With the help of functional magnetic resonance imaging (fMRI) studies, we have managed to demonstrate the effectiveness of mindfulness training in enhancing the cognitive control and have defined a particular pattern of regional brain activation consistently associated with mindful states [15]. By increasing attention, researchers believe mindful meditation (MT) can improve the core symptoms of ADHD that include task completion, self-regulation and impulse control. Neuroimaging studies have also determined the overlapping brain regions that are implicated in emotion dysregulation in ADHD and the changes associated with mindful meditation. The areas involved including the prefrontal cortex (including dorsal and ventromedial regions), hippocampus, and amygdala were associated with improvement in emotion regulation after mindfulness training. These areas are also involved in emotional functioning in individuals with ADHD diagnosis [16]. There is a rationale for the mindfulness-based approach to treatment for ADHD. Many studies have measured the impact of mindfulness training in experienced meditators and even a brief training with meditation yields improvements in attention [17]. For instance, one study assessed the impact of five days (20 minutes per day) of meditation against relaxation. The result of the study after treatment showed that the meditation group performed much better on group detection than the relaxation group during the attentional task. Similar findings have been reported following four days of meditation training (20 minutes per day) [18]. Brief mindfulness training has shown improvement in executive functioning, visuospatial processing, and working memory. Findings of this and others studies suggested that even four days of meditation training can enhance the ability to sustain attention [18-19]. Mindfulness-based cognitive therapy (MBCT) is a form of interventional therapy that combines cognitive behavioral therapy (CBT) with mindfulness based meditation. Mindfulness can be defined as paying attention in a particular way and staying focused and relaxed in the present moment. The best approach for MBCT is in a group format with eight weekly sessions each of 2.5 hours and then an off day (silent day). Such group-delivered therapies are often more cost-effective than individual treatment. Furthermore, MCBT has the additional benefit over stimulants of reducing anxiety, depression, and stress in addition to treating the core problem of inattention [20].

### Review of ADHD and mindfulness treatment studies

Several recent studies in adult ADHD samples provide promising preliminary support for mindfulness meditation training. There is emerging evidence that mindfulness meditation employed as a neurobehavioral intervention can help ADHD patients to regulate brain functioning and thereby improve the conscious direction of attention and emotional control [21].

We reviewed around 16 studies in recent years on the effects of mindfulness-based cognitive therapies in adults, the majority of which show a clear beneficial effect on ADHD symptoms in adults [13, 22, 23,24,25, 23, 26,27,28, 29,30,31,32, 20, 33,34]. Of 12 studies conducted within the last five years on the effectiveness of mindfulness-based cognitive therapies for ADHD in adults, all showed small-to-significant symptom reductions. A recent study by Cole, et al. in Geneva enrolled 49 adult ADHD patients with inadequate prior response to medication in a one-year program of individual therapy and weekly sessions of group therapy with different modules: mindfulness, emotion regulation, emotional tolerance, interpersonal effectiveness and impulsivity/hyperactivity and attention. Overall, the psychotherapeutic treatment resulted in significant improvements in almost all dimensions. The most important changes were observed for depression severity [Beck Depression Inventory (BDI)-II] (b=-0.30; p <0.0001), ADHD severity [ autism spectrum rating scales (ASRS) total score] (b=-0.16; p< 0.0001), and mindfulness skills [Kentucky inventory of mindfulness skills (KIMS) AwA] (b=0.21; p <0.0001), with moderate to large effect sizes. ADHD patients clearly showed a better pattern of response compared to control groups [34].

Another study conducted in 2015 reported that mindfulness-based cognitive therapy (MBCT) resulted in a significant reduction of ADHD symptoms and the symptom improvement was calculated both by investigators and by self-reported questionnaires [32]. The data clearly shows that mindfulness awareness practices improve attentional performance, affective symptoms, and quality of life in adult ADHD patients and should be considered as a useful complementary treatment in for adults with ADHD.

## Conclusions

Adult ADHD can have a huge personal and economic impact. There is strong evidence that medications can be effective in adults, decreasing the symptoms and their sequelae. However, there remains a great need for evidence-based psychosocial interventions to serve as adjunct or alternative treatment for individuals intolerant or not desiring pharmacotherapy. Mindfulness-based cognitive therapy is an effective intervention and can be administered in an economically-feasible group format. Some of the limitations of studies to date include small sample size, single center enrollment, lack of follow-up and lack of control groups. There is a clear need for further research in this area.
